# Predictors for the severe coronavirus disease 2019 (COVID-19) infection in patients with underlying liver disease: a retrospective analytical study in Iran

**DOI:** 10.1038/s41598-021-82721-3

**Published:** 2021-02-04

**Authors:** Mansour Bahardoust, Mohammad Heiat, Mehrdad Khodabandeh, Ashraf Karbasi, Zahra Bagheri-Hosseinabadi, Mohammad Hossein Ataee, Narjes Seidalian, Amirhossein Babazadeh, Shahram Agah, Mohammad Ali Abyazi

**Affiliations:** 1 Baqiyatallah Research Center for Gastroenterology and Liver Diseases, Baqiyatallah University of Medical Sciences, Tehran, Iran; 2 Department of Physical Medicine and Rehabilitation, Neuromusculoskeletal Research Center, Iran University of Medical Sciences, Tehran, Iran; 3 Department of Clinical Biochemistry, Faculty of Medicine, Rafsanjan University of Medical Sciences, Rafsanjan, Iran; 4 Applied Microbiology Research Center, Systems Biology and Poisonings Institute, Baqiyatallah University of Medical Sciences, Tehran, Iran; 5 Applied Virology Research Center, Baqiyatallah University of Medical Sciences, Tehran, Iran; 6 Student Research Committee, Baqiyatallah University of Medical Sciences, Tehran, Iran; 7 Colorectal Research Center, Iran University of Medical Sciences, Tehran, Iran

**Keywords:** Diseases, Infectious diseases

## Abstract

Risk factors for clinical outcomes of COVID-19 pneumonia have not yet been well established in patients with underlying liver diseases. Our study aimed to describe the clinical characteristics and outcomes of COVID-19 infection among patients with underlying liver diseases and determine the risk factors for severe COVID-19 among them. In a retrospective analytical study, 1002 patients with confirmed COVID-19 pneumonia were divided into two groups: patients with and without underlying liver diseases. The admission period was from 5 March to 14 May 2020. The prevalence of underlying conditions, Demographic data, clinical parameters, laboratory data, and participants' outcomes were evaluated. Logistic regression was used to estimate the predictive factors. Eighty-one (8%) of patients had underlying liver diseases. The frequencies of gastrointestinal symptoms such as diarrhea and vomiting were significantly higher among patients with liver diseases (48% vs. 25% and 46.1% vs. 30% respectively, both P < 0.05). Moreover, ALT and AST were significantly higher among patients with liver diseases (54.5 ± 45.6 vs. 37.1 ± 28.4, P = 0.013 and 41.4 ± 27.2 vs. 29.2 ± 24.3, P = 0.028, respectively). Additionally, the mortality rate was significantly high in patients with liver disease (12.4% vs. 7%, P = 0.018). We also observed that the parameters such as neutrophil to leukocyte ratio [Odds Ratio Adjusted (OR_Adj_) 1.81, 95% CI 1.21–3.11, P = 0.011] and blood group A (OR_Adj_ 1.59, 95% CI 1.15–2.11, P = 0.001) were associated with progression of symptoms of COVID-19. The presence of underlying liver diseases should be considered one of the poor prognostic factors for worse outcomes in patients with COVID-19.

## Introduction

The novel coronavirus, called SARS-CoV-2, which causes severe acute respiratory syndrome (COVID-19), was first reported in China in December 2019 and spread quickly worldwide^1^. Similar to the previous members of this family, COVID-19 commonly causes respiratory tract infection, which can be asymptomatic, mild, or severe. However, in some cases, it can cause a lethal condition. Although the mortality rate of COVID-19 is believed to be low, older adults and those with a chronic disease are at increased risk of severe symptoms, poorer prognosis, and even fatal conditions^1^. Furthermore, multi-organ damages are common among infected patients due to an inflammatory cascade induced by COVID-19. Heart failure, renal failure, acute respiratory distress syndrome (ARDS), liver damage, and shock can be the most important of these complications^2,3^.

Liver injury, which is often seen among severe cases of COVID-19^4,5^, is mostly accompanied by increased liver enzymes and bilirubin^6^. Although some studies have proven that COVID-19 can cause liver injury, the exact effects of background liver diseases on clinical outcomes of COVID-19 infection are not clear. One study showed no correlation between liver diseases and increased risk of COVID-19^7^. Moreover, the prognostic factors in COVID-19 patients with underlying liver diseases are not clear. Some studies have suggested that lab tests such as lymphopenia and high CRP levels are independently correlated with liver injury^8,9^. Yet, more extensive studies are needed to define the possible risk factors in patients with underlying liver diseases for developing severe COVID-19.

Our study aimed to describe the clinical characteristics, laboratory data and epidemiologic factors in COVID-19 patients with and without underlying liver disease and discuss the potential risk factors for developing severe symptoms in patients with underlying liver diseases.

## Materials and methods

This Retrospective study was approved by the Research Ethics Committee of Baqiyatallah University of Medical Sciences, Tehran, Iran (IR.BMSU.REC) with number code IR.BMSU.REC. 1399. 186. We obtained the patients’ informed consent to be allowed to use their medical information. The methods were carried out in accordance with the relevant guidelines and regulations.

In this retrospective analytical study from 5 March to 4 May 2020, we evaluated the records of 1002 cases with COVID-19 pneumonia who were admitted to our centers (Baqiyatallah and Rasoul-e-Akram Hospitals) in Tehran, Iran. The diagnosis of COVID-19 for all subjects was confirmed using real-time PCR and CT-scan. Written consent for research purposes was taken from patients at the time of diagnosis. According to the National Health Commission (Trial Version 5)^10^ and the guideline for the diagnosis and treatment of COVID-19 infection, all patients with positive PCR test and imaging feature in favor of COVID-19 were considered as infected cases. Besides, at the beginning of the admission, patients with respiratory rate > 30 breaths/min, SpO_2_ < 93% or PaO_2_/FiO_2_ ≤ 300 mmHg, were defined as severe and otherwise considered as mild cases.

The data collection checklist encompassed different characteristics such as demographic data including age, sex, educational level, smoking habits, trip history (in the last 2 weeks), and clinical features like initial symptoms, past medical history and comorbidities (e.g., hypertension, diabetes, cardiovascular diseases, and respiratory diseases), initial laboratory investigations including complete blood count, coagulation function, routine biochemical, and liver function tests data. We also collected the CT-scan features for all participants. The outcomes were defined as death, readmission, and duration of hospitalization. The records of subjects with considerable lacking were excluded. Participants were divided into two groups; (1) cases with liver diseases such as cirrhosis, grade II or higher fatty liver, and viral hepatitis, (2) cases without liver disease. The diagnosis of any liver diseases was confirmed via patients’ previous documents.

### Statistical analysis

The mean and standard deviation (SD) were conducted to describe continuous variables and qualitative data. The descriptive analysis was performed using frequencies. We used Kolmogorov–Smirnov and Shapiro–Wilk normality tests, P–P plot, and histogram to evaluate our study data's normality. For the continuous variables, a t-test was employed to analyze the mean difference in parametric and the Mann–Whitney U test in the non-parametric condition. The Chi-squared test (χ^2^ test) was conducted for the classified variables. Logistic regression analysis was use to evaluate the risk factors for severe COVID-19. Variables with P < 0.10 in the univariate analysis test were entered into a multivariate logistic regression analysis with the backward selection method. Two-tailed P-values < 0.05 were considered as statistically significant values. IBM SPSS Statistics 20.0 software (https://www.ibm.com/analytics/spss-statistics-software) software was used to analyze the data.

## Results

During the study period, 1002 patients with confirmed COVID-19 pneumonia were enrolled, among whom 81 patients (8%) had underlying liver disease. The mean age in liver diseases and non-liver diseases groups was 62.2 ± 14.9 and 58.5 ± 12.9 years, respectively. The male subjects were 56 (69%) in liver diseases and 609 (66.1%) in the non-liver diseases groups. There was no significant difference in age and sex between the two groups (both P-values > 0.05). Regarding the previous history of respiratory disease, no significant difference was observed for such a variable. The frequency of cases with severe symptoms was significantly higher in the liver diseases group (P-value = 0.011). Moreover, at the admission time, the frequency of patients with gastrointestinal complications such as diarrhea and vomiting were higher among the participants with liver diseases (P-value < 0.05). Table 1 shows a summary of the baseline characteristics and clinical history of the participants.

**Table 1:** Baseline characteristics of study population.

Variable	Liver diseases	Non-liver diseases	P-value
	Group (N: 81)	(N: 921)	
Age	62.2 ± 13.95	58.5 ± 12.9	0.41
**Sex**			0.72
Male	56 (69%)	609 (66.1%)	
Female	25 (31%)	312 (33.9%)	
**Exposure history (in the last 2 weeks)**			
Contact with patients	45 (55.5%)	444 (48.2%)	0.45
Go hospital	52 (64.1%)	590 (64%)	0.68
Public transportation	23 (28.4%)	230 (25%)	0.77
Current smoker	6 (7.4%)	42 (4.5%)	0.33
Hypertension	26 (32.1%)	276 (29.9%)	0.57
Diabetes	21 (26%)	219 (23.8%)	0.38
CVD	16 (19.7%)	166 (17.9%)	0.59
MI	3 (3.7%)	25 (2.7%)	0.18
Lung Diseases and COPD	18 (22.2%)	149 (16.2%)	0.11
Asthma	5 (6.1%)	43 (4.7%)	0.23
Kidney Diseases	5 (6.1%)	66 (7.1%)	0.46
Cancer	2 (2.4%)	17 (1.8%)	0.33
**Blood group**			
A	35 (43.2%)	258 (28%)	0.001
B	17 (20.9%)	231 (25%)	0.14
AB	8 (9.8%)	112 (12.3%)	0.26
O	21 (26.1%)	183 (34.7%)	0.048
**Classification COVID-19**			0.011
Mild	18 (22.2%)	553 (60%)	
Severe	63 (77.8%)	368 (40%)	

*MI* myocardial infarction, *COPD* chronic obstructive pulmonary disease, *CVD* cardiovascular disease.

Table 2 represents the laboratory and clinical features of the included cases. The presence of underlying liver diseases led to a higher mortality rate, which means poorer prognosis. The patients with underlying liver diseases significantly stayed longer at the hospital (94% vs. 68.7%, P = 0.021). During this period, the levels of alanine aminotransferase (ALT) and aspartate aminotransferase (AST) were significantly higher in patients with known liver diseases (54.5 ± 45.6 vs. 37.1 ± 28.4, 41.4 ± 27.2 vs. 29.2 ± 24.3, both P-value < 0.05). Furthermore, the patients with liver diseases showed degrees of coagulopathy since the INR in this group was significantly higher than the other group (1.9 ± 0.18 vs. 1.02 ± 0.09; P-value = 0.001).

**Table 2:** Clinical characteristics and initial symptoms and laboratory indices among patients with and without liver diseases.

Variable	Normal range	Liver diseases	Non-liver diseases	P-value
		Group (N: 81)	(N: 921)	
**Fever**	N (%)			0.015
≥ 38.5 °C		35 (43.2%)	175 (19%)	
< 38.5 °C		46 (56.8%)	746 (81%)	
Cough	N (%)	47 (58%)	552 (60%)	0.82
Chest pain	N (%)	20 (25%)	221 (24%)	0.51
Dizziness	N (%)	20 (25%)	313 (34%)	0.12
Headache	N (%)	26 (32%)	275 (29.8%)	0.59
Weakness	N (%)	47 (58%)	497 (54%)	0.42
Myalgia	N (%)	49 (60%)	485 (53.3%)	0.33
Diarrhea	N (%)	39 (48%)	230 (25%)	0.036
Vomiting	N (%)	37 (46.1%)	276 (30%)	0.033
Test sense	N (%)	13 (16%)	166 (18.1%)	0.31
**Laboratory findings, median (IQR)**				
Leucocytes (× 109/L)	4–10	9.522 ± 7.64	6.2 ± 3.01	0.016
Neutrophils (× 109/L)	02–07	61.17 ± 0.82	4.4 ± 0.75	0.043
Lymphocytes (× 109/L)	0.8–4	2.4 ± 1.94	2.01 ± 1.22	0.077
Platelets (× 109/L)	125–450	162.2 ± 56.2	155.65 ± 72.2	0.066
Hemoglobin (g/L)	12-18	15.1 ± 1.5	14.6 ± 1.6	0.081
Hematocrit (%)	38–50.8	44.15 ± 3.9	43.2 ± 4.5	0.19
**Blood biochemistry**				
Albumin (g/L)	40–55	45.5 ± 7.3	40.5 ± 6.1	0.031
ALT (U/L)	9–50	54.5 ± 45.6	37.1 ± 28.4	0.031
AST (U/L)	15–40	41.4 ± 27.2	29.2 ± 24.3	0.028
Total bilirubin (mmol/L)	0–26	10.2 ± 3.7	8.9 ± 4.3	0.15
Serum sodium (mmol/L)	137–147	136.6 ± 15.5	138.1 ± 18.9	0.55
Serum potassium (mmol/L)	3.5–5.3	3.55 ± 2.3	3.71 ± 2.6	0.48
BS(Mg/dL)	70–140	119.1 ± 24.3	111.2 ± 25.3	0.078
INR	0.85–1.15	1.9 ± 0.18	1.02 ± 0.09	0.001
CRP (mg/L)^d^	0–10	45.2 ± 32.1	39.2 ± 31.2	0.045
**Chest CT findings**	N (%)			
Mild conflict		7 (8.6%)	110 (12%)	0.059
Unilateral		11 (13.5%)	249 (27.1%)	0.001
Bilateral		32 (39.5%)	313 (34%)	0.22
Ground-glass opacity		31 (38.4%)	249 (26.9%)	0.042
**Clinical outcome**				
Increased hospital stay > 7 days	N (%)	76 (93.8%)	598 (65%)	0.021
Death	N (%)	10 (12.4%)	65 (7%)	0.018
Readmission	N (%)	8 (9.8%)	98 (10.6%)	0.42

*PCR* Polymerase chain reaction, *ALT* alanine aminotransferase, *AST* aspartate transaminase, *BS* blood sugar, *INR* international normalized ratio, *CRP* C-reactive protein.

### Risk factors of severe COVID-19 among the cases with liver disease

We conducted an adjusted logistic regression analysis to control confounders or collinearity to assess the factors associated with severe COVID-19 infection in patients with liver diseases. Variables with P-value < 0.10 in the univariate analysis test including age, the severity of COVID-19 taste sense, vomiting, diarrhea, blood group, leucocytes, neutrophils, lymphocytes, platelets, hemoglobin, albumin, ALT, AST, BS, INR, chest CT findings, hospital stay and death were entered into logistic regression analysis. As shown in Table 3, age ≥ 60 years, the higher level of neutrophil-to-lymphocyte ratio (NLR), blood group A and presence of any other comorbidity were predictable risk factors for the severity of COVID-19 in cases with liver diseases. Notably, after performing the same model on the cases without liver diseases, it was revealed that older ages and any other comorbidity were associated with a higher risk of severe symptoms of COVID-19. However, unlike patients with liver diseases, blood group A showed a protective effect (OR_Adj_ 0.67, 95% CI 0.39–0.96, P-value = 0.001).

**Table 3:** Multivariate logistic regression of factors associated with progression of disease in patients with underlying liver diseases and without underlying liver diseases.

Variable	Group					
	Liver diseases			Non-liver diseases		
	OR_Adj_	95% CI	P value	OR_Adj_	95% CI	P-value
Age ≥ 60 year	1.29	1.06–1.35	0.001	1.09	1.03–2.11	0.048
Comorbidities	1.32	1.09–3.12	0.011	1.12	1.03–3.55	0.023
Blood group (A)	1.59	1.15–2.11	0.001	0.67	0.39–0.96	0.001
Chest CT findings (unilateral)	0.42	0.12–0.76	0.023	0.72	0.41–1.58	0.068
NLR	1.81	1.21–3.11	0.011	1.02	0.66–2.97	0.28

*OR*_*Adj*_ Odds ratio adjusted, *95% CI* 95% confidence interval, *NLR* neutrophil-to-lymphocyte ratio.

## Discussion

This study showed that underlying liver diseases would lead to probable serious outcomes in COVID-19 patients. Our study showed that age ≥ 60 years, higher levels of NLR, blood group A and the presence of any other comorbidity were predictable risk factors for the severity of COVID-19 in the patients suffering from liver diseases. In contrast, in the non-liver diseases group, only two factors of age ≥ 60 years and the presence of any other comorbidity were associated with the severity of COVID-19. This is in line with the results of a meta-analysis conducted by Francesco et al.^11^. However, age ≥ 60 years was reported as a risk factor in both liver and non-liver diseases groups for the severity of COVID-19. It's OR was higher in liver diseases than non-liver diseases group (1.29 vs. 1.09). It is probably due to more disruption of innate and adaptive immunity in patients with liver diseases. Because such diseases interfere with the production of complements, antimicrobial peptides, and cytokines, moreover, the antigen presentation to the immune system will be disturbed in liver diseases^12^.

Several comorbidities (e.g., hypertension, diabetes, cardiovascular diseases, and respiratory diseases) are associated with poorer outcomes in infected individuals^13^. However, liver-related comorbidities were not common among the patients with COVID-19 pneumonia and their frequency was about 3%^14^. Although liver enzyme abnormality was common in patients, it barely resulted in a serious liver injury^4,5,13^. This can imply that the liver is not a target organ for SARS-CoV-2. The very low expression of the ACE2 receptor in hepatocytes and bile duct epithelium^15,16^ may explain the small influence of viral infection in the liver. Our study also revealed that patients with liver diseases are more susceptible to COVID-19 than cases without comorbidity. Based on our study results, the presence of any other comorbidity in both groups was significantly associated with the severity of COVID-19. However, the OR for this risk factor was higher in liver diseases than the non-liver diseases group (1.32 vs. 1.12). It can be justified by the synergistic interaction between liver diseases and comorbidities in this group. The liver diseases themselves could affect other parts like the circulatory system (enlarges blood vessels) as well as lung and kidney, hepatopulmonary and hepatorenal syndromes, for instance. On the other hand, the association of metabolic syndrome and glucose intolerance in fatty liver disease can significantly influence the heart. These findings could be more prominent in elders, explaining more OR for the severity of symptoms in the liver diseases group older than sixty and suffering from any other comorbidity. Moreover, the administration of hepatotoxic drugs could be the other coexisting mechanism^14^. Although most patients with underlying liver diseases had mild infections, the frequency of severe outcomes was significantly higher among them. Therefore, the presence of abnormalities in the liver may accelerate the progression of the illness.

Patients with chronic liver diseases, such as cirrhosis, are vulnerable to infections because of their immunocompromised state^12,17^. Besides, the SARS-CoV-2 virus damages the lymphocytes, especially T cells^18,19^, which leads to impairment in a patient’s immune system function and makes them more susceptible to infections. Furthermore, a higher level of NLR may increase the risk of bacterial infections^20^. A meta-analysis demonstrated that the increased level of NLR is associated with poorer outcomes in patients with COVID-19^21^. Consistent with the previous reports, we observed that this marker's higher level is directly proportional to the severity of disease in the liver diseases group. However, no significant correlation was observed for NLR levels in patients with liver disease. This observation may indicate the high probability of bacterial superinfection among these populations.

The association of blood group type with several viral infections has been proven^9,22^. The results of our study revealed that the patients with blood group A had a higher risk of COVID-19 progression. While in the non-liver diseases group, blood group A had a protective role. This finding is consistent with the previous studies in this respect. Zhao et al. showed that blood group A subjects are more likely to get COVID-19 pneumonia than the subjects with non-A blood groups^23^. In an in vivo setting, Guillon et al. demonstrated that the human natural anti-A antibodies would prevent the interaction between the ACE2 receptor and SARS-CoV-2, offering protection for subjects who possess such antibodies^24^.

Our study had some strengths and weak points. The main strong point of this study was a considerably large number of included participants with COVID-19 infection. The study's main weakness was the small number of patients with liver diseases, which did not allow several analyses. Due to limited medical resources during this pandemic, patients with severe COVID-19 pneumonia were more likely to be hospitalized.

## Conclusion

This study's findings demonstrated that the presence of any chronic liver disease is associated with poorer outcomes in a population with COVID-19.
